# Action spectrum for photoperiodic control of thyroid-stimulating hormone in Japanese quail (*Coturnix japonica*)

**DOI:** 10.1371/journal.pone.0222106

**Published:** 2019-09-11

**Authors:** Yusuke Nakane, Ai Shinomiya, Wataru Ota, Keisuke Ikegami, Tsuyoshi Shimmura, Sho-Ichi Higashi, Yasuhiro Kamei, Takashi Yoshimura

**Affiliations:** 1 Institute of Transformative Bio-molecules (WPI-ITbM), Nagoya University, Nagoya, Japan; 2 Laboratory of Animal Integrative Physiology, Graduate School of Bioagricultural Sciences, Nagoya University, Nagoya, Japan; 3 Division of Seasonal Biology, National Institute for Basic Biology, Okazaki, Japan; 4 Department of Physiology, School of Medicine, Aichi Medical University, Nagakute, Japan; 5 Department of Agriculture, Tokyo University of Agriculture and Technology, Fuchu Japan; 6 Spectrography and Bioimaging Facility, National Institute for Basic Biology, Okazaki, Japan; 7 Avian Bioscience Research Center, Graduate School of Bioagricultural Sciences, Nagoya University, Nagoya, Japan; University of Texas Southwestern Medical Center, UNITED STATES

## Abstract

At higher latitudes, vertebrates exhibit a seasonal cycle of reproduction in response to changes in day-length, referred to as photoperiodism. Extended day-length induces thyroid-stimulating hormone in the pars tuberalis of the pituitary gland. This hormone triggers the local activation of thyroid hormone in the mediobasal hypothalamus and eventually induces gonadal development. In avian species, light information associated with day-length is detected through photoreceptors located in deep-brain regions. Within these regions, the expressions of multiple photoreceptive molecules, opsins, have been observed. However, even though the Japanese quail is an excellent model for photoperiodism because of its robust and significant seasonal responses in reproduction, a comprehensive understanding of photoreceptors in the quail brain remains undeveloped. In this study, we initially analyzed an action spectrum using photoperiodically induced expression of the beta subunit genes of thyroid-stimulating hormone in quail. Among seven wavelengths examined, we detected maximum sensitivity of the action spectrum at 500 nm. The low value for goodness of fit in the alignment with a template of retinal1-based photopigment, assuming a spectrum associated with a single opsin, proposed the possible involvement of multiple opsins rather than a single opsin. Analysis of gene expression in the septal region and hypothalamus, regions hypothesized to be photosensitive in quail, revealed mRNA expression of a mammal-like melanopsin in the infundibular nucleus within the mediobasal hypothalamus. However, no significant diurnal changes were observed for genes in the infundibular nucleus. *Xenopus*-like melanopsin, a further isoform of melanopsin in birds, was detected in neither the septal region nor the infundibular nucleus. These results suggest that the mammal-like melanopsin expressed in the infundibular nucleus within the mediobasal hypothalamus could be candidate deep-brain photoreceptive molecule in Japanese quail. Investigation of the functional involvement of mammal-like melanopsin-expressing cells in photoperiodism will be required for further conclusions.

## Introduction

At higher latitudes, day-length and ambient temperature undergo marked annual variations. To adapt to these changing environments, vertebrates show flexible physiological and behavioral responses, a phenomenon known as photoperiodism. Through anticipating these seasonal changes and mating at a specific time of year, vertebrates tend to give birth to offspring within a limited period during the spring and summer months. This control of seasonal reproduction is a consequence of the ability of these animals to detect changes in day-length.

Owing to its robust and significant photoperiodic responses, the Japanese quail (*Coturnix japonica*) has been used in a number of studies on photoperiodism. Levels of the plasma luteinizing hormone (LH) in these birds begin to increase shortly after exposure to a light period that exceeds 12 h [1]. Several studies using Japanese quail have focused on two brain regions, the mediobasal hypothalamus (MBH) and pars tuberalis of the pituitary gland (PT), as the centers of the photoperiodic response [1,2]. In birds, an extended day-length induces the synthesis and secretion of thyroid-stimulating hormone (TSH) in the PT. TSH derived from the PT, acting via TSH receptors located within the ependymal cells (EC) lining the third ventricle (3V) of the MBH, triggers the expression of type 2 deiodinase (DIO2), which converts thyroid prohormone thyroxin (T4) to the bioactive 3,5,3'-triiodothyronine (T3) [1]. This local activation of thyroid hormone in the MBH has been suggested to control the secretion of gonadotropin-releasing hormone (GnRH) from the median eminence. This release is photoperiodically triggered by morphological changes among GnRH-nerve terminals and glial cells [3]. The key roles of TSH and DIO2 in reproduction has been illustrated not only in mammals [4–6] but also in fish [7].

Despite numerous findings relating to the signal transduction cascade regulating photoperiodism, the molecular and cellular characteristics of the photoreceptors that detect an elongated photoperiod are still not fully understood. Non-mammalian vertebrates have extra-retinal photoreceptors in the pineal organ and deep-brain regions. However, in birds, the eyes and pineal organ have been found to be non-essential for photoperiodism [8,9]. von Frisch first suggested the role of photosensitive brain regions in regulating changes in the skin color of the European minnow (*Phoxinus phoxinus*) [10]. Local illumination of the brain using luminescent beads or light fibers has revealed that deep-brain photoreceptors mediate gonadal development in avian species such as ducks [11], sparrows [12], and the Japanese quail [13–15], and photoreceptive molecules referred to as opsins, including rhodopsin [16–18], vertebrate ancient (VA)-opsin [19], melanopsin [20,21], opsin 5 [22–24], and opsin 3 [25], have been reported to be expressed in various regions of the avian brain.

Previous studies have proposed opsin 5 and VA-opsin as candidate molecules that may mediate the reception of light information in the photoperiodic response of quail. Opsin 5 is a short wavelength-sensitive opsin [22,23,26], and short wavelengths of light ranging from 300 to 450 nm are known to trigger photoperiodic testicular growth in quail [22]. Knock down of opsin 5 in the paraventricular organ (PVO) within the MBH has been shown to suppress the photoperiodic induction of mRNA encoding the beta subunit of TSH (*TSHB*) in the PT [24]. VA-opsin, which was first isolated from Atlantic salmon (*Salmo salar*) [27–29], has been characterized as a photopigment with a peak sensitivity ranging from ~460 nm to 500 nm [28,30,31]. Expression of this photopigment has also been confirmed in the quail brain [19]. VA-opsin-like immunoreactive neurons are distributed throughout the paraventricular nucleus (PVN), anterior medialis hypothalami (AM), nucleus supraopticus pars ventralis (SOv), and nucleus magnocellularis preopticus pars ventralis (MPOv) within the anterior hypothalamus [19]. It has been suggested that these neurons not only project into the PT but also directly control GnRH secretion, because several populations of these neurons co-express VA-opsin- and GnRH-like immunoreactivities [19,32]. Using action spectrum techniques based on circulating levels of LH, Foster and colleagues predicted the involvement of an opsin-based photopigment with a λ_max_ at ~492 nm in quail [33,34]. Re-analysis of the predicted spectrum using nonlinear regression revealed that the λ_max_ occurs at ~483 nm [19], thereby indicating that the peak sensitivity of the spectrum in quail corresponds closely to that of chicken VA-opsin (λ_max_ at ~ 491 nm) [31].

However, in spite of these previous findings, given that a variety of opsins are observed in different avian brains, the involvement of deep brain-expressed opsins in regulating the photoperiodic increase in *TSHB* has yet to be conclusively determined. In this study, we constructed and evaluated an action spectrum for expression of photoperiodically controlled *TSHB* in the PT of Japanese quail, utilizing an Okazaki Large Spectrograph (OLS), which is a broad-spectrum exposure system that can produce a range of wavelengths from 250 to 1,000 nm. On the basis of the action spectrum results, we identified mammal-like melanopsin (*OPN4m*), expressed in the infundibular nucleus (IN) within the MBH, as a candidate deep-brain photoreceptive molecule in Japanese quail.

## Results

### The transmittance pattern of monochromatic light penetrating the quail hypothalamus

**Fig 1:**
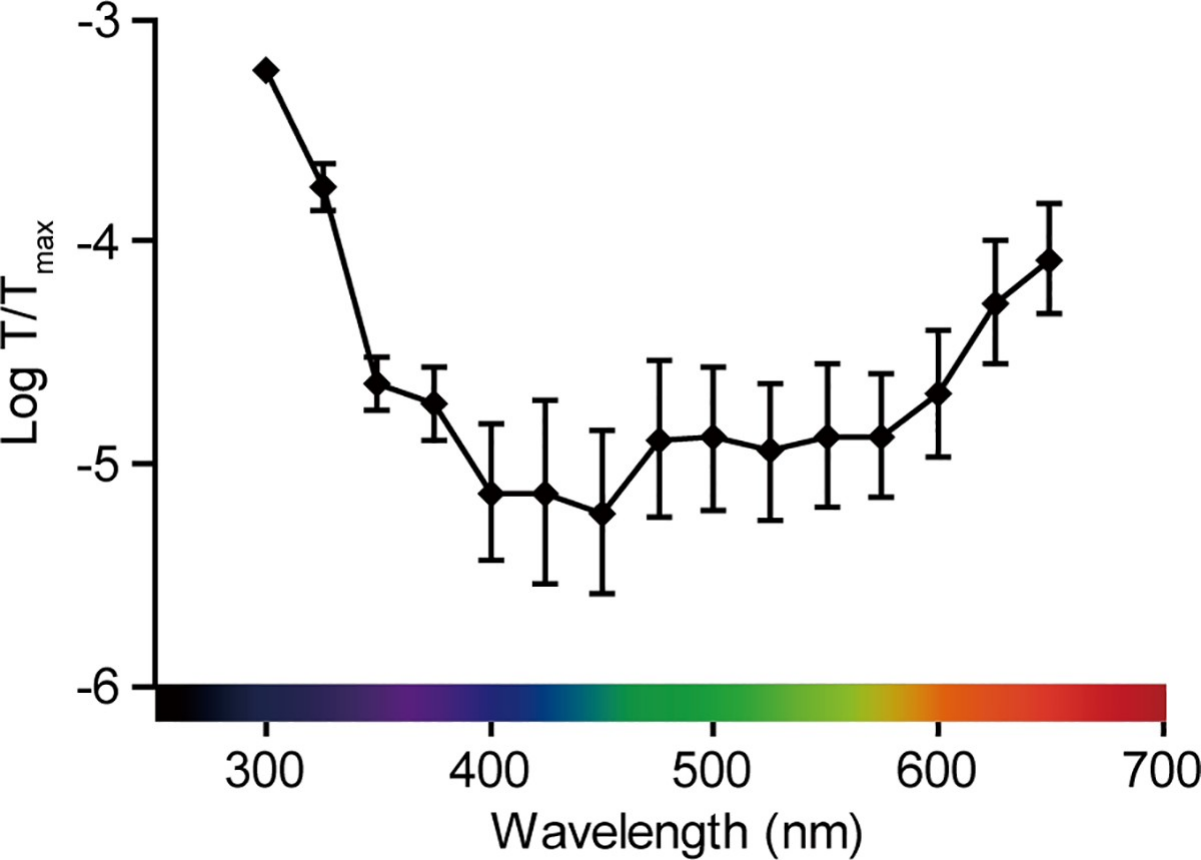
Spectral characteristics of the transmittance of various wavelengths of light reaching the quail hypothalamus. Relative transmittance pattern (T/T_max_) of various wavelengths of light penetrating the feathers, skin, skull, and brain tissue are indicated. Each point represents the mean ±SEM (n = 3).

Differences are observed in the relative transmittance of monochromatic light penetrating the feathers, skin, and brain tissue depending on the wavelength of transmittance, which is attributable to the characteristic absorption spectra of water, protein, lipid, bone, and other effectors [33–36]). Heads lacking the lower jaws were cut below the hypothalamus and placed on a black-taped slide glass containing a central window. The top of the birds’ heads were illuminated with different wavelengths of monochromatic light, ranging from 300 to 650 nm, and a luminance meter was then placed just below the window. The spectral transmittance (T/T_max_) of the light reaching the hypothalamus was calculated by measuring the luminance without the bird’s head (T_max_) and at the ventral border of the hypothalamus (T) (Table 1). Beneath the hypothalamus, the transmittance declined from 300 nm to 450 nm before gradually increasing to 650 nm (Fig 1).

**Table 1:** Light intensity without a bird's head (T_max_) and at the hypothalamic level (T) at different wavelengths.

Wavelength (nm)	Light intensity (μW/cm^2^)		T/T_max_
	T_max_	T (*n = 3*)	
300	0.655	3.80.E-04	5.80.E-04
325	4.30	9.68.E-04	2.25.E-04
350	45.5	1.29.E-03	2.83.E-05
375	86.0	2.28.E-03	2.65.E-05
400	135	2.16.E-03	1.60.E-05
425	142	3.34.E-03	2.35.E-05
450	176	2.80.E-03	1.60.E-05
475	147	5.10.E-03	3.47.E-05
500	125	3.79.E-03	3.04.E-05
525	141	3.57.E-03	2.54.E-05
550	139	4.45.E-03	3.20.E-05
575	128	3.48.E-03	2.71.E-05
600	80.5	3.44.E-03	4.28.E-05
625	80.0	8.00.E-03	1.00.E-04
650	65.5	9.75.E-03	1.49.E-04

### Spectral light sensitivity of photoreceptors responsible for the photoperiodic induction of *TSHB*

**Fig 2:**
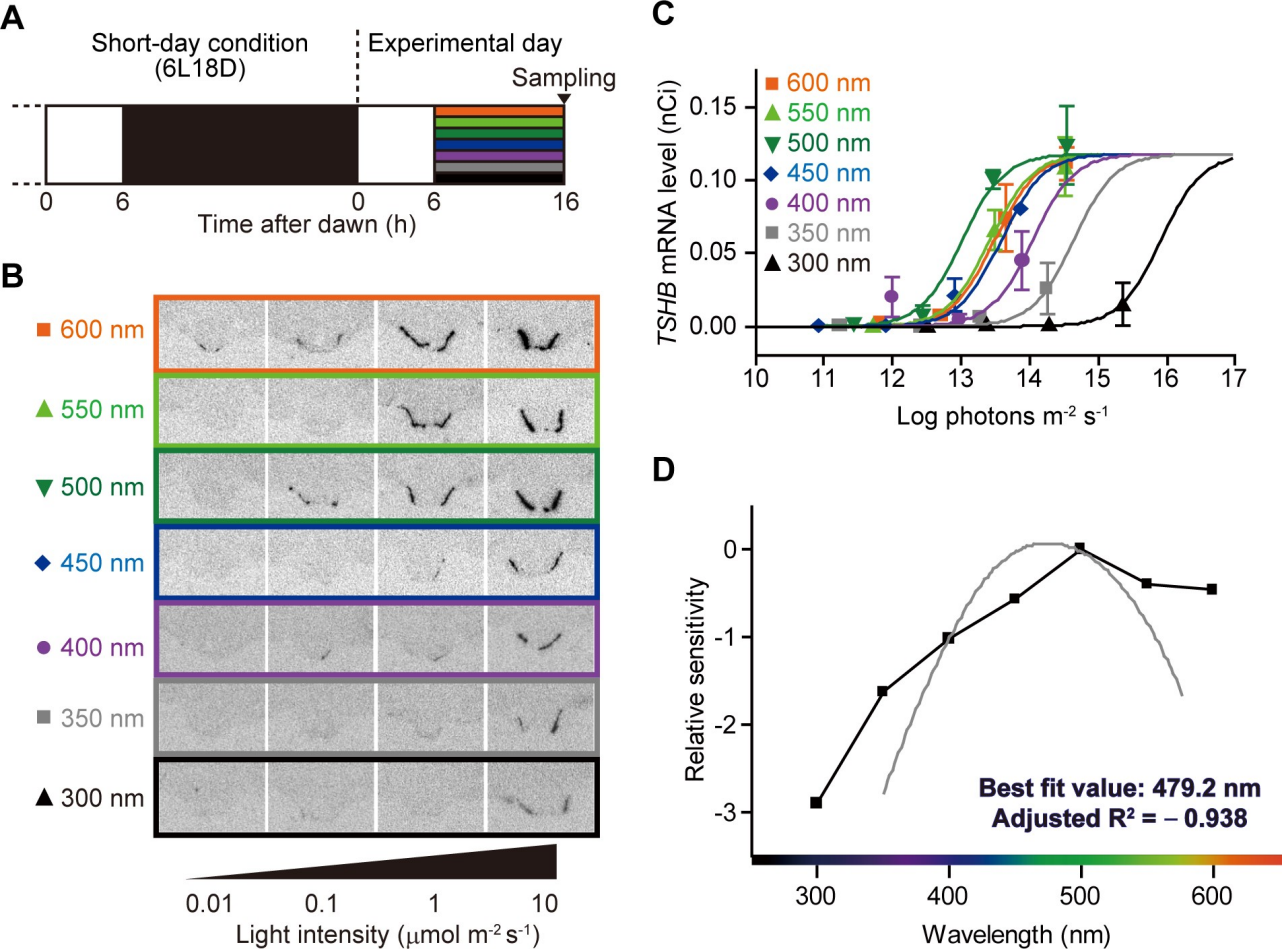
The action spectrum using the photoperiodically induced beta subunit of thyroid-stimulating hormone. (**A**) Schedule for light exposure and sampling. Eye-patched birds lacking pineal organ and maintained under short-day conditions (6 h:18 h light/dark cycle: 6L18D) were given a long-day stimulus by extending the 6-h light period by 10 h with light of seven different wavelengths. (**B**) The effects of four intensities of light at each wavelength on the expressions of photoperiodically induced mRNA encoding the beta subunit of thyroid-stimulating hormone (*TSHB*) in the pars tuberalis of the pituitary gland were evaluated by *in situ* hybridization. (**C**) Each expression level of *TSHB* was plotted to examine the dependence of photoperiodic responses on irradiance with monochromatic lights of seven different wavelengths. Each light intensity (photons m^2^ s^-1^) was adjusted to the light intensity at the deep-brain region level using the percentage of T/T_max_. Each point represents the mean ± SEM (n = 4). (**D**) The action spectrum for photoperiodic *TSHB* induction. The half-saturation constant (EC_50_) derived from sigmoidal fits of the light intensity–response curves were plotted against wavelength. The action spectrum was then fitted with a curve for retinal1-based photopigment using the least-squares method. Peak sensitivity was approximately 479.2 nm with a low value for the goodness of fit (adjusted R^2^ = -0.938).

In birds, light is detected by the eyes and pineal organ. Although there have been several reports that neither of these organs plays a major role in testicular development in birds [8,9], we should not exclude the possibility that eyes and the pineal organ are involved in photoperiodic responses directly, indirectly, or in unexpected ways, given that both organs are functional photoreceptors. Therefore, prior to exposure to light of wavelengths, we surgically removed the pineal organs from a total of 112 quails. During the 6-h white light period on the day of the experiment, the eyes of all birds were covered with eye patches. The pinealectomized and eye-patched birds maintained under the short-day condition were exposed to a long-day stimulus by extending the 6-h light period by 10 h. During the supplementary 10-h light exposure, the birds were illuminated with light of a range of intensities (0.01, 0.1, 1, 10 μmol m^-2^ s^-1^) at seven different wavelengths emitted from an OLS (Fig 2A). After the birds had been subjected to 16 h of light, their brains were collected to evaluate the expression of *TSHB* in the PT. This is because photoperiodic induction of *TSHB* in the PT peaks at 16 h after dawn [1]. We found that each of the seven wavelengths of light examined in this study induced the expression of *TSHB* in the PT, with the degree of induction being dependent on light intensity (Fig 2B). Following adjustment of the light intensity for each wavelength reaching the hypothalamus via transmittance (Fig 1 and Table 1), we plotted the expression level of photoinduced *TSHB* in the PT against the photons of each wavelength reaching the hypothalamus (Fig 2C). The light intensity–response relationship of the expression level of photoinduced *TSHB* in response to each wavelength light is shown separately in S1 Fig.

To evaluate the effects of light of different wavelengths on the induction of photoperiodic *TSHB* expression in the PT of pinealectomized and eye-patched birds, an action spectrum was plotted using the half-saturation constant (EC_50_) of the light intensity–response relationship and the seven wavelengths (S1 Fig). The action spectrum based on photoperiodically induced *TSHB* expression indicated that its peak sensitivity (λ_max_) lies at 500 nm (Fig 2D). The plot was aligned to the best-fit template of retinal1-based photopigment, assuming that a single opsin would be involved in the action spectrum [37,38]. Although the fitted curve indicated a maximal sensitivity at ~479.2 nm, we failed to detect a high coefficient of correlation for fitting the template to the spectrum (adjusted R^2^ = -0.938) (Fig 2D)

### Expression of melanopsin in the deep-brain regions

We found that the retinal1-based photopigment template did not align appropriately with the constructed spectrum, which was wider than the template (Fig 2D). Cone opsins, rhodopsin, and melanopsin contribute to the spectral sensitivity that mediates the circadian photoreceptors [39,40]. Previously, it has been shown that CBA/J (*rd/rd*) mice, which express melanopsin but are deficient in cone opsins and rhodopsin, exhibit an action spectrum for circadian entrainment that differs from that of mice with all three types of photoreceptors intact [38]. The discrepancy between the action spectrum and the template shown in Fig 2D suggested the possibility that multiple types of photoreceptive molecules rather than a single type are involved in the action spectrum for the photoperiodic induction of *TSHB* in quails. Consistent with this deduction, it has been proposed that at least two types of opsins (VA-opsin and opsin 5) are involved in the photoperiodic gonadal development of quails [19,22,24,32]. In the present study, we failed to detect the distinctive signal of the *OPNVA* mRNA coding for VA-opsin in the anterior hypothalamus of male birds under short-day conditions (S2 Fig). Although the spectral sensitivity of rhodopsin with λ_max_ at ~500 nm is similar to the maximum sensitivity of the action spectrum established in the present study, we also failed to detect mRNA coding for rhodopsin in regions of the quail brain in which its immunoreactivities have been observed in other avian species (S2 Fig) [16–18]. Therefore, we could hypothesize the expressions of other candidate photoreceptive molecules with predicted sensitivities ranging between ~470 to 520 nm.

**Fig 3:**
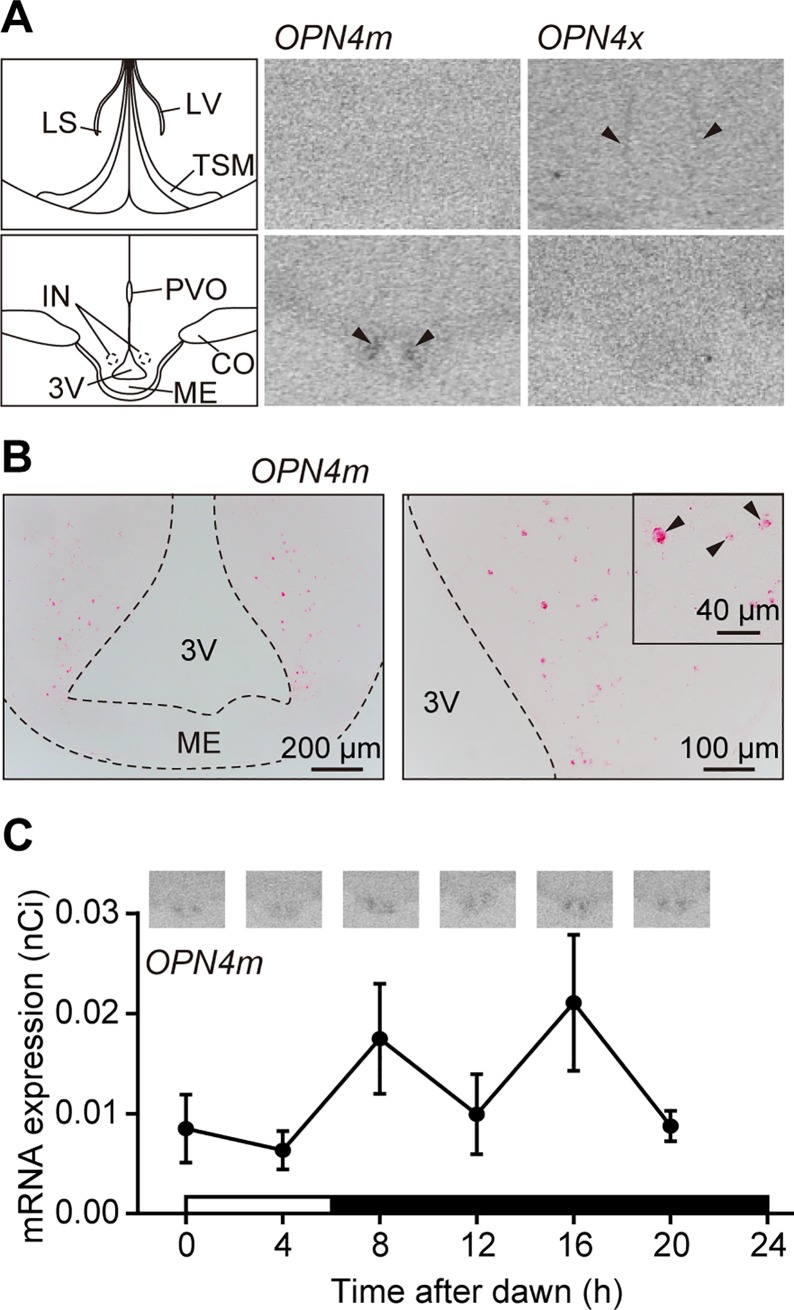
Expressions of mammal-like and *Xenopus*-like melanopsins in the quail brain. (**A**) The expression of mRNA encoding mammal-like melanopsin (*OPN4m*) was detected in the infundibular nucleus (IN) within the mediobasal hypothalamus (MBH) (arrowheads in the lower row); however, *OPN4m* was not detected in the septal region. Faint expression of *Xenopus*-like melanopsin (*OPN4x*) mRNA was detected in the septal region (arrowheads in the upper raw) but was not detected in the MBH. (**B**) Expression analysis of both melanopsins based on high-sensitivity *in situ* hybridization revealed the expression of *OPN4m* in the IN (arrowheads in a high-magnification image). (**C**) The JTK_CYCLE algorithm revealed a pattern of OP*N4m* expression in the IN that lacked a distinctive periodicity (adjusted p-value: 1.0, Benjamini–Hochberg q-value: 1.0, period: 20, Phase 12). Each point represents the mean ± SEM (n = 3). CO: optic chiasm, IN: infundibular nucleus, LS: lateral septum, LV: lateral ventricle, ME: median eminence, PVO: paraventricular organ, 3V: third ventricle.

We went on to examine the expression of melanopsin (which has a peak sensitivity at ~480 nm) in the septal region and hypothalamus, which are candidate sites for the photosensitive brain regions in quail [13–15]. In non-mammalian vertebrates, melanopsin has two isoforms, namely, mammal-like melanopsin (OPN4m) and *Xenopus*-like melanopsin (OPN4x) [41]. These two isoforms have been isolated in chickens and found to be blue light-sensitive opsins [42]. Given that previous studies have failed to detect the expression of melanopsin mRNA in the septal region and hypothalamus [22], in the present study, we increased the number of probes for each melanopsin and performed *in situ* hybridization using radioisotope-labeled probes. Gene expression analysis revealed expression of *OPN4m* in the IN within the MBH and faint expression of *OPN4x* in the septal region (Fig 3A). Owing to low image resolution, we re-examined the results using another highly sensitive *in situ* hybridization method, RNAscope [43]. We accordingly detected the expression of *OPN4m* in the IN (Fig 3B), although no expression of *OPN4x* was detected in the septal region (S3 Fig). We subsequently performed a quantitative analysis of the expression pattern of *OPN4m* in the IN by performing *in situ* hybridization with radioisotope-labeled probes, and found that expression in the IN did not show a prominent periodicity based on cycling parameter predictions using the JTK_CYCLE algorithm [44] (Fig 3C). However, it is notable that we did detect *OPN4x* in the pineal organ (S4 Fig), which is consistent with the findings of previous studies using chickens and turkeys [20,21,45]. In addition, we found that expression of *OPN4x* and *OPN4m* in the retina was localized in the ganglion cell layer (GCL), the outer half of the inner nuclear layer (INL), and a small subsection of the inner half of the INL (S5 Fig). The *DapB* gene, which encodes the enzyme dihydrodipicolinate reductase from the SMY strain of *Bacillus subtilis*, was used as a negative control probe; however, this enzyme was not detected in the tissues examined (S6 Fig).

## Discussion

In this study, we analyzed the characteristics of spectral transmittance reaching the hypothalamus in the brains of quails (Fig 1 and Table 1). The distinctive spectral transmittances we observed are essentially comparable with those reported in previous studies [33–36] (S7 Fig). Nevertheless, we cannot exclude the possibility that the observed spectral transmittances might include a certain range of error probability, given that the characteristic absorption spectra of water, protein, lipid, bone, and other effectors in living animals may differ from those in dead animals. Previously, it has been demonstrated that long-day exposure to UV-B and UV-A for 2 weeks resulted in the development of testes in pinealectomized and eye-patched quail [22]. In the present study, we exposed pinealectomized and eye-patched quail to light of a range of different wavelengths (Fig 2) and found that a single, long-day stimulus with any given wavelength from 300 nm to 600 nm is sufficient to trigger the expression of *TSHB* in the PT (Fig 2B). On the basis of a plotted action spectrum for photoperiodically induced *TSHB* in the PT, we determined a maximum sensitivity at 500 nm, which is consistent with previously reported action spectra based on plasma LH induction, testicular development, and cloacal gland size [33,34,36] (S7 Fig).

Although the retinal1-based photopigment template predicted a peak sensitivity at ~479.2 nm, a wavelength close to the spectrum based on circulating LH level (λ_max_ = 483 nm) [19], we found that the goodness of fit was significantly low (Fig 2D). Avian brains express a variety of opsins, and if multiple opsins play roles in mediating photophysiological processes such as photoperiodism and circadian photoentrainment, the characteristics of their action spectra (e.g., λ_max_ and shape) could be modified depending on the peak sensitivities and contributions of the different opsins. The poor fit obtained using the retinal1-based template in this study could be attributable to the contribution of the short wavelength-sensitive opsin 5, because the effect of shorter wavelengths of light on the photoinduction of *TSHB* is greater than the effect predicted using the retinal1-based template (Fig 2D). We therefore tried to analyze the action spectrum by multi-Gaussian curve fitting based on Python and SciPy. According to the analysis, two curves could be fitted to the action spectrum, and their peak sensitivities were at ~369.0 and ~522.8 nm, respectively. The two peak sensitivities were shifted from the current single peak sensitivity (at ~479.2 nm). However, these curves did not appear to fit properly because of a few data points in this data set (S8 Fig).

Previous studies based on local illumination using glass fibers have proposed that photosensitivity of the IN is associated with the induction of gonadal development in quail [14,15]. To date, however, there have been no studies that have examined the expressions of reported photosensitive molecules in the IN of quail. In the present study, we detected *OPN4m* mRNA as a candidate deep-brain photoreceptor in the IN (Fig 3). Previous studies have shown that rhodopsin and opsin 5 are expressed in the cerebrospinal fluid (CSF)-contacting neurons [16,17,22,23], which cytologically resemble developing photoreceptive cells in the retina and pineal organ [46]. Furthermore, electrophysiological studies have indicated that opsin 5-expressing CSF-contacting neurons in the paraventricular organ (PVO) of quail can be intrinsically photosensitive [24]. These results indicate that photosensitivity is a feature of CSF-contacting neurons in the quail brain. Interestingly, CSF-contacting neurons are also distributed in the IN of quail [47,48]. It would therefore be interesting to evaluate the intrinsic photosensitivity of neurons expressing OPN4m in the IN and determine whether OPN4m is expressed in CSF-contacting neurons in the IN. Opsin 5-expressing nerve fibers are distributed in the external layer of the median eminence, adjacent to the PT, and studies using a neuron tracer have revealed that the PVO in which opsin 5-expressing CSF-contacting neurons are localized, project directly into the adjacent area to the PT. Accordingly, it would of interest to examine the projection of OPN4m-expressing neurons.

*OPN4x* in the hypothalamic premammillary nucleus (PMM) of turkeys has previously been shown to be diurnally expressed, and it has been proposed that this expression pattern is associated with the activity of dopamine and melatonin, which are co-expressed in *OPN4x*-positive neurons [21], It has, however, been suggested that the PMM is redundant with respect photoperiodism in female turkeys [49]. In the present study, we failed to detect statistically significant periodicity in the expression of *OPN4m* in the IN of quails (Fig 3C). In the quail brain, the galanin- and serotonin-positive CSF-contacting neurons are localized in the IN as well as in the PVO. Further, it has been observed that the number of serotonin-positive neurons differs between day and night, although no comparable significant difference has been detected in the number of galanin-positive neurons. Interestingly, both serotonin- and galanin-positive fibers have been observed in the external layer of the median eminence adjacent to the PT [48]. It would therefore be interesting to investigate the co-localization of these neurotransmitters and OPN4m protein to determine whether OPN4m-expressing cells can transmit photo-signal information to the PT.

It has been suggested that VA-opsin is involved in photoperiodic-associated gonadal development in quails [19,32]. In the present study, however, we failed to detect *OPNVA* mRNA in the anterior hypothalamus of male birds maintained under short-day conditions (S2 Fig). Recently, it has been observed that expression levels of long wavelength-sensitive cone opsin undergo marked changes between summer and winter in the retina of medaka fish [50], thereby indicating that environmental factors such as temperature and day-length affects the expressions of opsins. Accordingly, we cannot exclude the possibility that the expression levels and/or numbers of *OPNVA*-expressing neurons were lower than the level of detection in our *in situ* hybridization analysis of quails maintained under short-day conditions, although further analysis is required in this regard. We also failed to detect mRNA coding for rhodopsin in regions of the quail brain where its immunoreactivities have been observed in other avian species (S2 Fig) [16–18]. Previous immunohistochemical studies have, however, failed to detect rhodopsin (which has a peak sensitivity at ~500 nm) in the quail brain [51,52].

Deep-brain photoreceptors have been studied in a range of avian species, including Japanese quails, chickens, turkeys, doves, pigeons, sparrows, and ducks, and these studies have not only established the presence of photoreceptors in avian brain and their involvement in photoperiodism, but also revealed a diversity in the distribution of photoreceptive molecules among avian species. Further detailed expression and functional analyses of opsins in avian species may contribute to elucidating how these multiple photoreceptors orchestrate the light information associated with photoperiodism.

## Materials and methods

### Animals

Four-week-old male Japanese quails (*Coturnix japonica*) were purchased from a local supplier. Birds were housed in bird cages (n = 5 per cage) placed in light-tight boxes (W136.5 cm × D45 cm × H42 cm) at a temperature of 23°C ± ±1°C. Water and food were provided *ad libitum*.

Birds were maintained under short-day (SD) condition (6 h:18 h light/dark cycle, ~200 lux during light period) until 8 weeks of age. Mature birds older than 8 weeks raised under the SD conditions were used for all experiments, with the exception of the *in situ* hybridization analysis using RNAscope. Mature male birds older than 8 weeks raised under the long-day conditions (14 h:10 h light/dark cycle) were purchased from the Nagoya University Graduate School of the Bioagricultural Sciences Avian Bioscience Research Center, Japan and used in RNAscope analysis. Food and water were provided *ad libitum*. Animals were treated in accordance with the guidelines of Nagoya University and the National Institutes of Natural Sciences, Japan. All experimental protocols were approved by the Animal Experiment Committee of Nagoya University and the National Institutes of Natural Sciences, Japan. Note that there were no animals showing any adverse clinical signs such as 20% loss in body weight within 7 days in all animal experiments. Minimal numbers of birds were used and all efforts were made to avoid any adverse effects.

### Spectral transmittance of light through brain tissues

Given that the transmittance of light of different wavelengths to the deep-brain region is not equal, we assessed spectral transmittance. Male birds maintained under SD conditions were decapitated, and heads lacking the lower jaws were cut below the hypothalamus and placed on a black-taped slide glass containing a central window (6 × 6 mm) (S9 Fig) Exposure to light was performed by using an Okazaki large spectrograph (OLS) at the National Institute for Basic Biology (NIBB). Elimination of stray light from crevices between the head and slide glass was achieved by covering with a black-colored adhesive. A 30-kW Xenon arc lamp mounted in the spectrograph emits monochromatic light ranging from 250 to 1000 nm onto its 10 m focal curve. Each monochromatic light projected from the OLS was introduced from the top of the bird’s head. A UDT 81 OPTOMETER luminance meter (United Detector Technology Co., Culver City, CA, USA) was placed immediately beneath the window. Luminance was also measured in the absence of a head to represent maximum transmittance (T_max_) and at the ventral border of the hypothalamus (T).

### Light exposure for action spectrum determination

Four- to eight-week-old birds maintained under the SD condition were anesthetized with inhalation of isoflurane and fixed on a stereotaxic instrument (MODEL900; David Kopf Instruments, Tujunga, CA, USA). The pineal organ was removed surgically through a hole (5 mm diameter) created in the lambda of the skull using fine forceps (1184007; Fine Science Tools, North Vancouvers, BC, Canada). The head skin was sutured after surgery. All surgical wounds had completely healed and were covered with feathers by the day of the experiment using light of different wavelengths. During the light period of the experimental day, both eyes in the pinealectomized birds were shielded from light using eye-patches prepared by cutting the adhesive piece of a Band-Aid plaster (8 mm in diameter), and pasting black tape on the non-adhesive side. After feathers from the area surrounding the eye had been removed, rubber cement was applied around the eye and around the edge of the patch, prior to placing it over the eye. On the experimental day, the 6-hour light period was extended by 10 hours using light of seven different wavelengths (300, 350, 400, 450, 500, 550, and 600 nm) and four light intensities (0.01, 0.1, 1, and 10 μmol/m^2^/s). At 16 hours after the start of the light period, birds were decapitated and the brains were immediately frozen by storage at -80°C. As in the spectral transmittance experiment, each monochromatic light at each of the four light intensities was projected from the OLS and introduced via the top of the bird’s head (S9 Fig).

### *In situ* hybridization

Non-perfused frozen brain sections (20 μm in thickness) were prepared using a CM3050S cryostat (Leica Biosystems, Wetzlar, Germany). The sections were examined using ^33^P-labeled oligonucleotide probes as previously described [53]. Hybridization was carried out overnight at 42°C, after which two high-stringency post-hybridization washes were performed at 55°C. The sections were air dried and exposed to BioMax MR film (Eastman Kodak Company, Rochester, NY, USA). The probe sequences used in this analysis are listed in S1 Table. We designed each probe sequence whose similarity was at most 79% among 13 opsins identified in birds and ~ 94% between chicken and quail. The optical densities of the signals for *TSHB* and ^14^C standards (American Radiolabeled Chemicals, Inc., St. Louis, MO, USA) were quantified using Multi gauge V3.0 software (Fujifilm, Tokyo, Japan). Birds were perfused with 4% paraformaldehyde and paraffin sections (8 μm in thickness) were prepared using an RM2235 microtome (Leica Biosystems, Wetzlar, Germany) for *in situ* hybridization using an RNAscope assay kit (Advanced Cell Diagnostics, Inc., Newark, CA, USA). Gene expression analysis using RNAscope was performed in accordance with the standard protocol for RNAscope. All probes used for this analysis were purchased from Advanced Cell Diagnostics, Inc. (Newark, CA, USA). A probe for *OPN4m* (C/N: 449251) was prepared based on the NM_001044653 sequence, whereas an *OPN4x* probe (C/N: 449161) was prepared based on the NM_204625 sequence. *DapB* coding for dihydrodipicolinate reductase (C/N: 310043) was used as a negative control probe following the RNAscope assay kit protocol.

### Data analysis

Light intensity was adjusted using the T/T_max_ ratio, and a light intensity–response curve was fitted with a sigmoidal curve using Graph Pad Prism 4 (MDF, Tokyo, Japan). The action spectrum was generated by plotting the EC_50_ for each wavelength. To assess the peak of the action spectrum, the equation for retinal-1 [37] was fitted by the least-squares method using Graph Pad Prism 4 [22]. Multi-Gaussian curve was fitted to the action spectrum using Python (v3.7.4) and scipy.optimize.curve_fit in SpiPy (v1.3.0).

## Supporting information

S1 Fig Irradiance–response curve for each of the different wavelengths of light. Each point represents mean ± SEM (n = 4). Goodness of fit shows high coefficient of correlation for each fitted curve.
(TIF)

S2 Fig Analysis of the expression of the *OPNVA* mRNA encoding vertebrate ancient (VA)-opsin and *RHO* mRNA coding rhodopsin in the quail brain. Despite signal enhancement by increasing the number of anti-sense probes,no *OPNVA* mRNA signals were detected in the anterior hypothalamus of quail brains. Similar, no distinct signals for *RHO* mRNA were observed in the septal region and mediobasal hypothalamus.
AC: anterior commissure, AM: anterior medialis hypothalami, CO: optic chiasma, IN: infundibular nucleus, PVN: paraventricular nucleus, TSM: septopalliomesencephalic tract, LS: lateral septum, LV: lateral ventricle, ME: median eminence, PVO: paraventricular organ, 3V: third ventricle.
(TIF)

S3 Fig Analysis of the expression of *Xenopus*-like melanopsin (*OPN4x*) mRNA in the septal region of quail brains. (**A**) No signals for *OPN4x* mRNA were observed in the septal region using the highly sensitive RNAscope *in situ* hybridization technique. (**B**) High-magnification images of areas around the lateral ventricles in (**A**).
TSM: septopalliomesencephalic tract, LV: lateral ventricle.
(TIF)

S4 Fig Analysis of the expression of mammal- and *Xenopus*-like melanopsin mRNAs in the pineal organ of quail brains. Expression of *Xenopus*-like melanopsin (*OPN4x*) mRNA was detected in the pineal organ (arrow), as has previously been reported in chickens [19]. In contrast, no expression of mammal-like melanopsin (*OPN4m*) mRNA was detected in the pineal organ.
(TIF)

S5 Fig Expressions of mammal- and *Xenopus*-like melanopsin mRNAs in the retina of quails. Distinctive *OPN4m* and *OPN4x* mRNA signals were detected in the ganglion cell layer (arrows), the inner half of the inner nuclear layer (arrowheads) and the outer half of the inner nuclear layer.
GCL: ganglion cell layer, IPL: inner plexus layer, INL: inner nuclear layer, OPL: outer plexus layer, ONL: outer nuclear layer, RPE: retinal pigment epithelium.
(TIF)

S6 Fig Analysis of the expression of *DapB* mRNA as a negative control. No signals of negative control *DapB* mRNA were detected in the septal region (**A**), the infundibular nucleus (**B**), or the retina (**C**) of quails.
3V: third ventricle, GCL: ganglion cell layer, IPL: inner plexus layer, INL: inner nuclear layer, OPL: outer plexus layer, ONL: outer nuclear layer; RPE: retinal pigment epithelium.
(TIF)

S7 Fig Comparison of the datasets for the spectral transmittance and action spectrum among three independent studies. The two independent datasets of spectral transmittance reaching the hypothalamus in quail (**A**) and the action spectra for photoperiodic responses (**B**) based on the plasma level of luteinizing hormone (LH) (upper, red) (33,34), testes weight (middle green solid line), cloacal gland (middle green dotted line) (36), were re-drawn and compared with those based on photoperiodically induced beta subunit of thyroid-stimulating hormone (*TSHB*) in the pars tuberalis of the pituitary gland (PT) (lower, black).
(TIF)

S8 Fig Multi-Gaussian curve fitting analysis of the action spectrum. The action spectrum was analyzed by multi-Gaussian curve fitting based on Python and SciPy. The analysis predicted that two curves could be fitted to it, and their peak sensitivities were at approx. 369.0 and approx. 522.8 nm, respectively.
(TIF)

S9 Fig The schematic diagrams of the spectral transmittance experiment and the light exposure experiment for action spectrum. (**A**) the schematic diagrams of the spectral transmittance experiment. Heads lacking the lower jaws were cut below the hypothalamus and placed on a black-taped slide glass containing a window (6 × 6 mm) in its middle position. A UDT 81 OPTOMETER luminance meter was placed just under the window. Each monochromatic light projected from the OLS was introduced from the top of the bird’s head.
(**B**) Four birds were placed facing each other. Each monochromatic light with various light intensities was introduced from the top of the four bird’s head.
(TIF)

S1 Table Sequences of the 33P-labeled riboprobes used in *in-situ* hybridization. (XLSX)
