# Supplementary figures and images for: Action spectrum for photoperiodic control of thyroid-stimulating hormone in Japanese quail (Coturnix japonica)
Source: PLoS One. 2019 Sep 11;14(9):e0222106. doi: 10.1371/journal.pone.0222106 (PMC6738599; doi:10.1371/journal.pone.0222106)

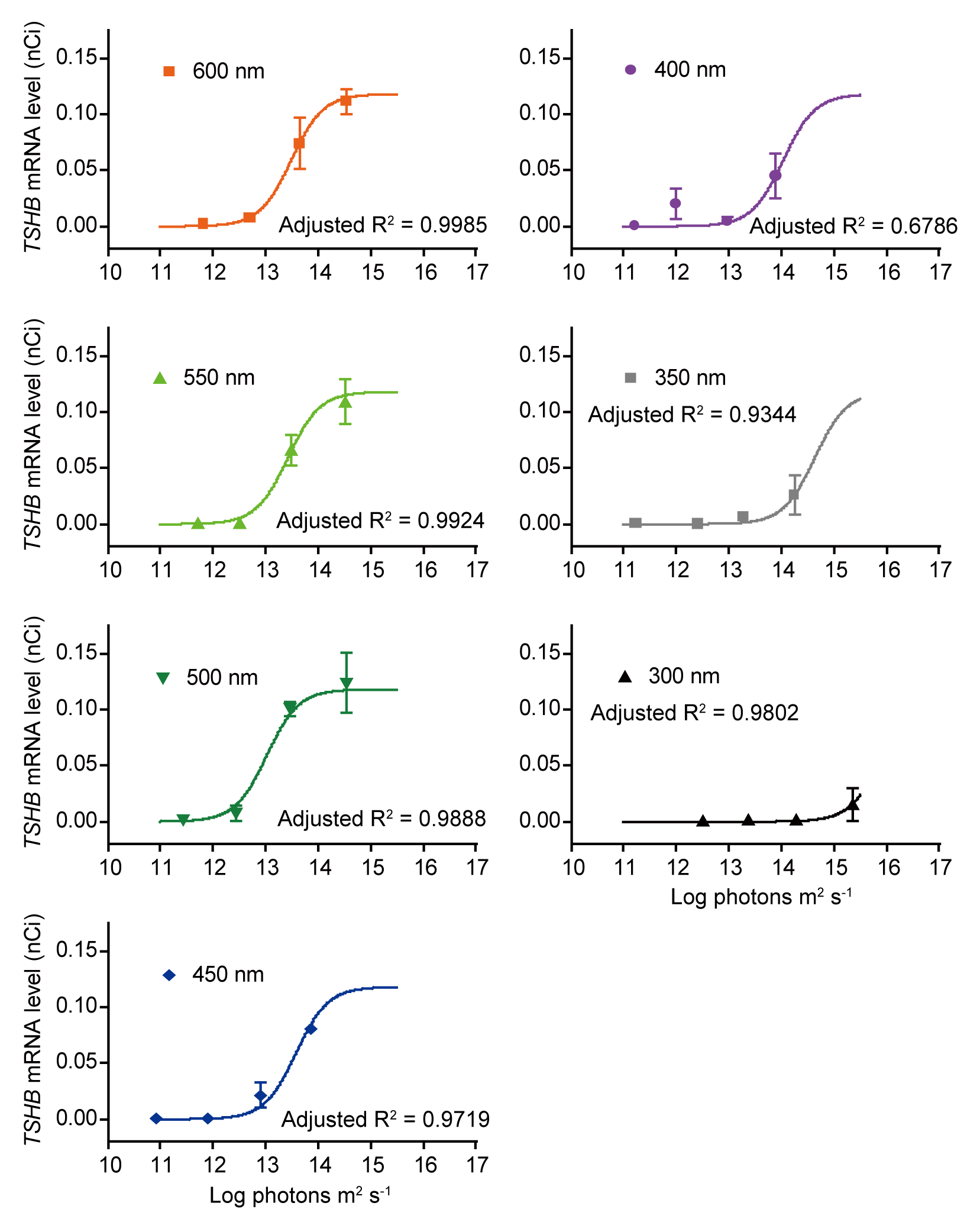

Supplement: S1 Fig — Each point represents mean ± SEM (n = 4). Goodness of fit shows high coefficient of correlation for each fitted curve. (TIF) [file pone.0222106.s001.tif]

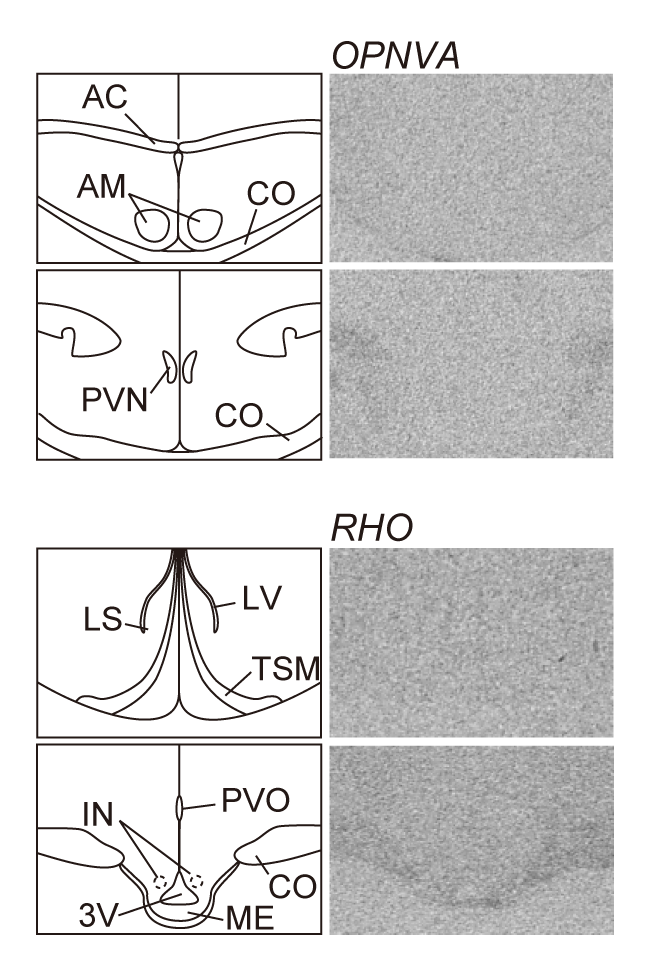

Supplement: S2 Fig — Despite signal enhancement by increasing the number of anti-sense probes,no OPNVA mRNA signals were detected in the anterior hypothalamus of quail brains. Similar, no distinct signals for RHO mRNA were observed in the septal region and mediobasal hypothalamus. AC: anterior commissure, AM: anterior medialis hypothalami, CO: optic chiasma, IN: infundibular nucleus, PVN: paraventricular nucleus, TSM: septopalliomesencephalic tract, LS: lateral septum, LV: lateral ventricle, ME: median eminence, PVO: paraventricular organ, 3V: third ventricle. (TIF) [file pone.0222106.s002.tif]

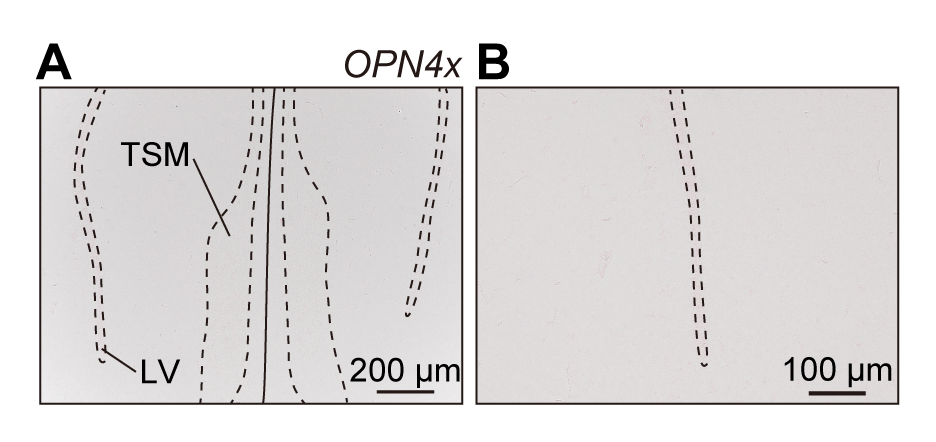

Supplement: S3 Fig — (A) No signals for OPN4x mRNA were observed in the septal region using the highly sensitive RNAscope in situ hybridization technique. (B) High-magnification images of areas around the lateral ventricles in (A). TSM: septopalliomesencephalic tract, LV: lateral ventricle. (TIF) [file pone.0222106.s003.tif]

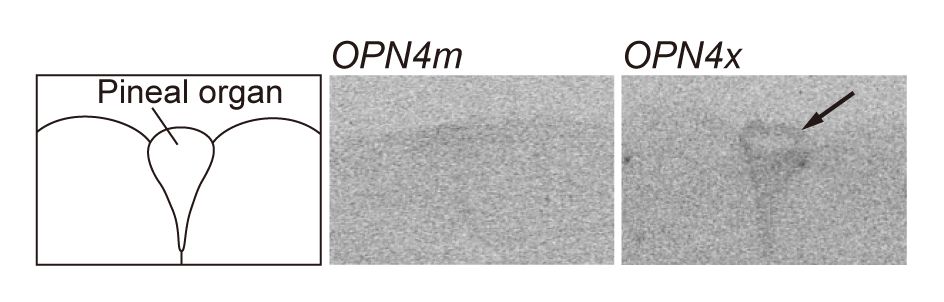

Supplement: S4 Fig — Expression of Xenopus-like melanopsin (OPN4x) mRNA was detected in the pineal organ (arrow), as has previously been reported in chickens [19]. In contrast, no expression of mammal-like melanopsin (OPN4m) mRNA was detected in the pineal organ. (TIF) [file pone.0222106.s004.tif]

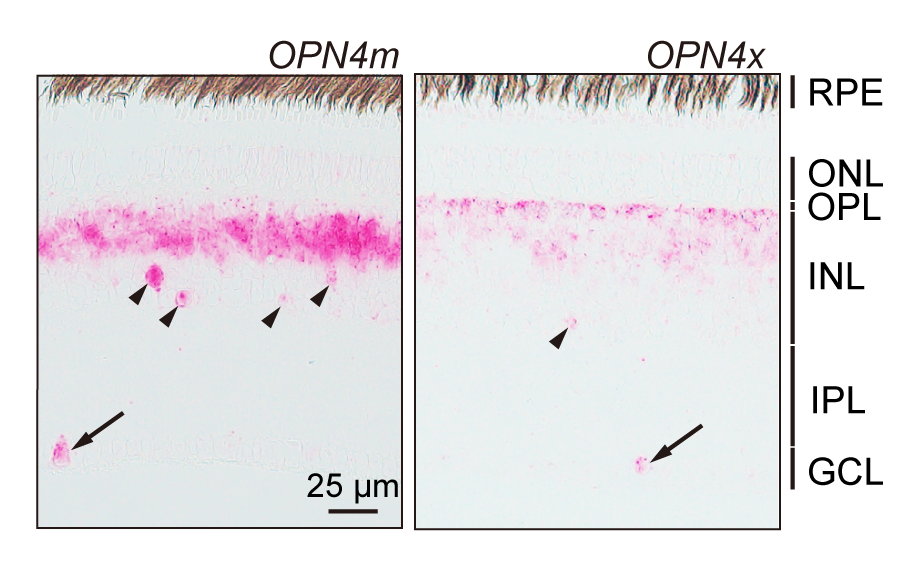

Supplement: S5 Fig — Distinctive OPN4m and OPN4x mRNA signals were detected in the ganglion cell layer (arrows), the inner half of the inner nuclear layer (arrowheads) and the outer half of the inner nuclear layer. GCL: ganglion cell layer, IPL: inner plexus layer, INL: inner nuclear layer, OPL: outer plexus layer, ONL: outer nuclear layer, RPE: retinal pigment epithelium. (TIF) [file pone.0222106.s005.tif]

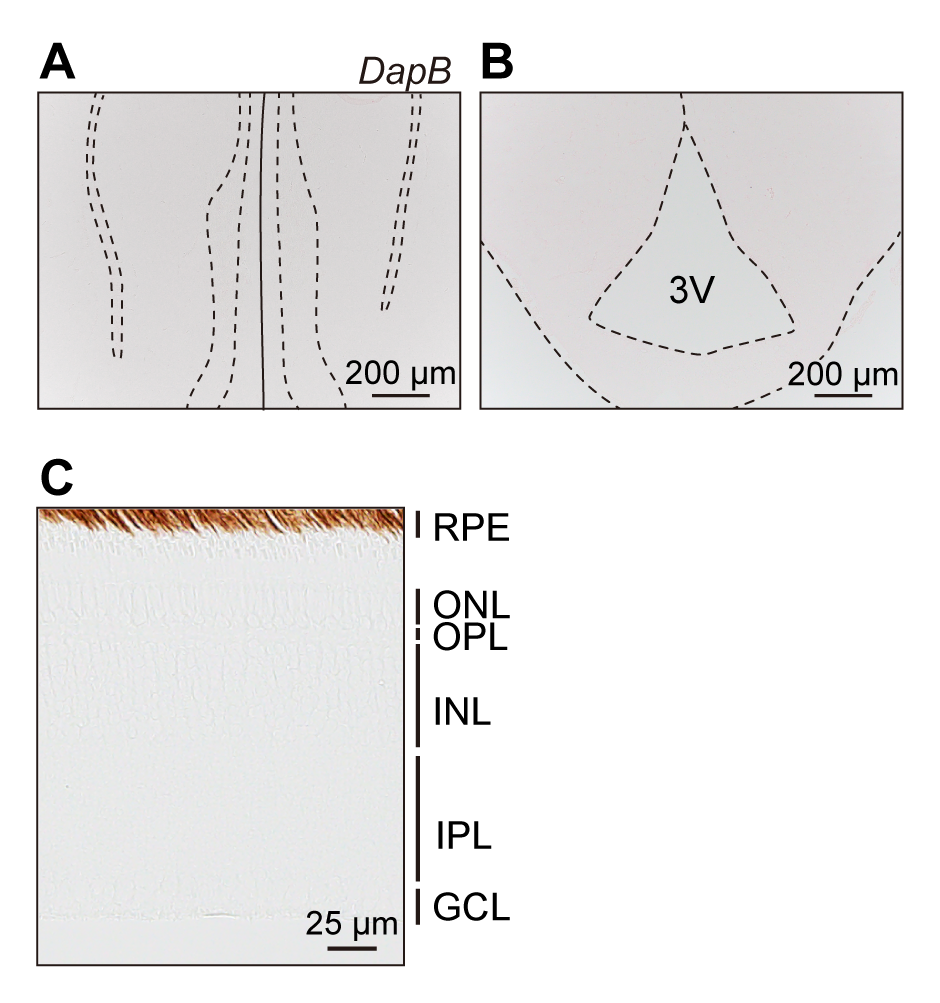

Supplement: S6 Fig — No signals of negative control DapB mRNA were detected in the septal region (A), the infundibular nucleus (B), or the retina (C) of quails. 3V: third ventricle, GCL: ganglion cell layer, IPL: inner plexus layer, INL: inner nuclear layer, OPL: outer plexus layer, ONL: outer nuclear layer; RPE: retinal pigment epithelium. (TIF) [file pone.0222106.s006.tif]

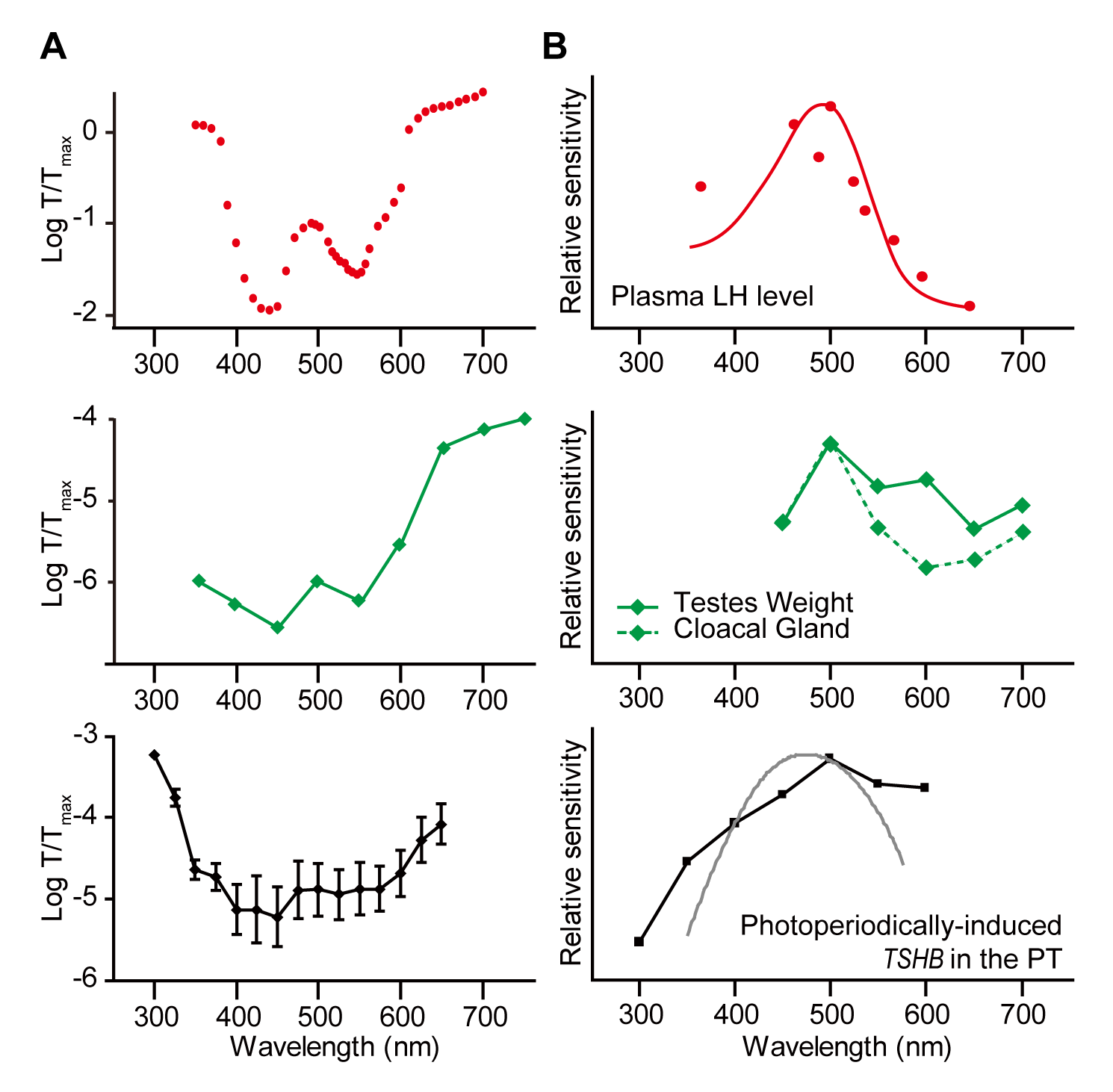

Supplement: S7 Fig — The two independent datasets of spectral transmittance reaching the hypothalamus in quail (A) and the action spectra for photoperiodic responses (B) based on the plasma level of luteinizing hormone (LH) (upper, red) (33,34), testes weight (middle green solid line), cloacal gland (middle green dotted line) (36), were re-drawn and compared with those based on photoperiodically induced beta subunit of thyroid-stimulating hormone (TSHB) in the pars tuberalis of the pituitary gland (PT) (lower, black). (TIF) [file pone.0222106.s007.tif]

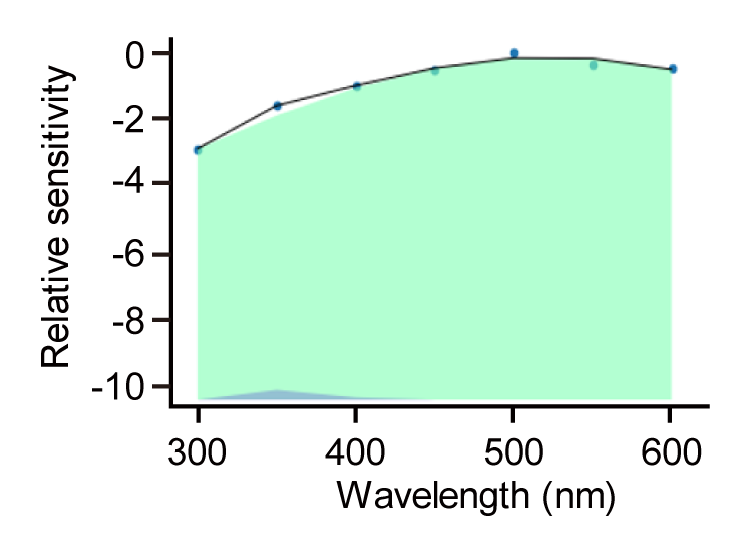

Supplement: S8 Fig — The action spectrum was analyzed by multi-Gaussian curve fitting based on Python and SciPy. The analysis predicted that two curves could be fitted to it, and their peak sensitivities were at approx. 369.0 and approx. 522.8 nm, respectively. (TIF) [file pone.0222106.s008.tif]

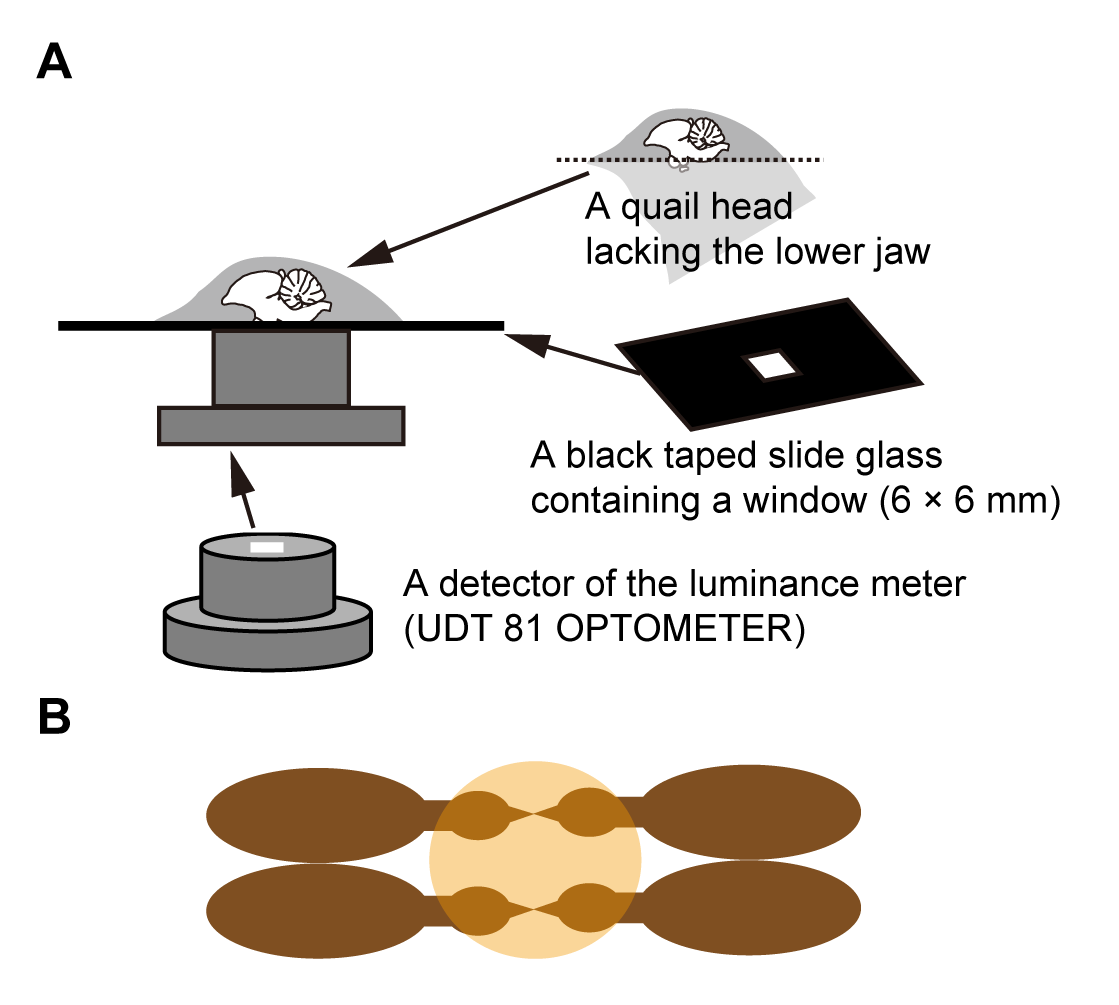

Supplement: S9 Fig — (A) the schematic diagrams of the spectral transmittance experiment. Heads lacking the lower jaws were cut below the hypothalamus and placed on a black-taped slide glass containing a window (6 × 6 mm) in its middle position. A UDT 81 OPTOMETER luminance meter was placed just under the window. Each monochromatic light projected from the OLS was introduced from the top of the bird’s head. (B) Four birds were placed facing each other. Each monochromatic light with various light intensities was introduced from the top of the four bird’s head. (TIF) [file pone.0222106.s009.tif]
